# Serum CA125 level is a good prognostic indicator in lung cancer.

**DOI:** 10.1038/bjc.1990.355

**Published:** 1990-10

**Authors:** Y. Kimura, T. Fujii, K. Hamamoto, N. Miyagawa, M. Kataoka, A. Iio

**Affiliations:** Department of Radiology, Ehime University School of Medicine, Japan.

## Abstract

The serum CA125 level was determined by a one-step immunoradiometric assay method in patients with lung cancer. Increased serum CA125 levels were observed in 37.8% of patients with squamous cell cancer, in 30.0% of those with adenocarcinoma and in 60.0% of those with small call cancer. Most patients with increased serum CA125 levels were in stages 3 or 4. Patients with pleural effusions or ascites showed high serum CA125 levels. The survival time was significantly shorter in patients with increased serum CA125 levels than in those within normal limits. Among patients with advanced disease (stages 3 and 4), an increased serum CA125 level was again a poor prognostic factor (P less than 0.01). The existence of a pleural effusion did not correlate with the survival time. We conclude that CA125 is a good indicator of disease extent and serum levels correlate to the length of survival.


*Br. J. Cancer* (1990), **62**, 676-678 © Macmillan Press Ltd., 1990

### Serum CA125 level is a good prognostic indicator in lung cancer

Y. Kimura, T. Fujii, K. Hamamoto, N. Miyagawa, M. Kataoka & A. Iio

*Department of Radiology, Ehime University School of Medicine, Shitsukawa, Shigenobu-chou, Onsen-gun, Ehime 791-02, Japan.*

**Summary** The serum CA125 level was determined by a one-step immunoradiometric assay method in patients with lung cancer. Increased serum CA125 levels were observed in 37.8% of patients with squamous cell cancer, in 30.0% of those with adenocarcinoma and in 60.0% of those with small call cancer. Most patients with increased serum CA125 levels were in stages 3 or 4. Patients with pleural effusions or ascites showed high serum CA125 levels. The survival time was significantly shorter in patients with increased serum CA125 levels than in those within normal limits. Among patients with advanced disease (stages 3 and 4), an increased serum CA125 level was again a poor prognostic factor ($P<0.01$). The existence of a pleural effusion did not correlate with the survival time. We conclude that CA125 is a good indicator of disease extent and serum levels correlate to the length of survival.

Owing to the recent progress in immunology, many monoclonal antibodies against various malignant tissues and their products have been developed and some of them are now in clinical use. One of these is OC125, which is obtained by immunising mice with ovarian cancer cell lines (Bast *et al.*, 1981). The antigen recognised by this antibody, CA125, has been proved to show increased serum levels in a high percentage of patients with ovarian cancer and endometriosis (Bast *et al.*, 1983; Kabawat *et al.*, 1983*a*; Baumah *et al.*, 1987; Fleuren *et al.*, 1987; Takahashi *et al.*, 1986). Recently, other antibodies, designated 130-20 and 145-9 have been prepared by immunising mice with the lung adenocarcinoma cell line PC-9 (Matsuoka *et al.*, 1987; Kunimatsu *et al.*, 1988). These antibodies recognise the same molecule as does OC125, but the epitopes which are recognised are different for each antibody. As the antibodies 13-20 and 145-9 were raised against a pulmonary adenocarcinoma cell line, it seemed possible that CA125 could also be a tumour marker for lung adenocarcinoma. We examined serum CA125 levels in patients with lung cancer by one-step immunoradiometric assay, and found, contrary to the first hypothesis, that this antigen was not specific only to lung adenocarcinoma but it also reflected the extent of lung cancers of various types including adenocarcinoma and their prognosis.

### Materials and methods

### *Assay kit*

The assay kit for CA125 was kindly donated by the Daiichi Radioisotope Laboratory (Tokyo, Japan). The assay method was a one-step immunoradiometric assay using $^{125}$I-labelled antibody 130-20 solution and latex beads coated with antibody 145-9. The correlation between CA125 levels measured by the current method and those measured by the conventional method (ELSA CA125 kit, CIS, Saclay, France) was $y = 0.886x + 2.405$, $r = 0.931$, $n = 477$.

### *Subjects*

Blood samples were collected from 752 normal subjects in our institute and related hospitals. Serum samples obtained from 95 untreated patients with lung cancer and 43 patients with benign lung diseases from 1982 and 1987 were stored at −20°C until examination.

Correspondence: Y. Kimura.
Received 27 March 1990; and in revised form 31 May 1990.

### *Statistical analysis*

Differences of the CA125 level by sex and age in normal subjects were estimated by Student's $t$ test. The proportions of patients in various groups with the elevated marker were estimated by the $\chi^2$ test. The survival was calculated from the time of blood sampling. The survival rates were estimated by the Kaplan-Meier method and were compared using the generalised Wilcoxon test.

### Results

### *Normal range of serum CA125 using the one-step immunoradiometric assay kit*

CA125 levels ranged from 2.7 to 87.6 U ml$^{-1}$ (mean ± s.d. 15.3 ± 9.9). There existed a significant difference between the serum CA125 level of males and females. These were 9.6 ± 5.1 U ml$^{-1}$ and 17.3 ± 10.5 U ml$^{-1}$, respectively ($P<0.01$) (Figure 1). Among the females, there existed a significant difference between the serum CA125 levels of younger subjects (younger than 50) and older subjects ($P<0.01$). This may be due to the changes of menstrual activity around the age of 50, as the serum CA125 levels were higher in menstruating women and lower after menopause (Table I).

According to these results, we determined the cut-off values as 20 U ml$^{-1}$ for males and 38 U ml$^{-1}$ for females.

**Figure 1** Serum CA125 levels of normal subjects. The cut-off value for males is 20 U ml$^{-1}$ and that for females is 38 U ml$^{-1}$.

**Table I** Serum CA125 levels of normal females

| | $n$ | Mean ($U ml^{-1}$) | s.d. | Range |
|---|---|---|---|---|
| Oestrogenic phase | 20 | 16.8 | 5.9 | 8.1–30.4 |
| Ovulatory phase | 9 | 10.9 | 2.4 | 8.2–14.9 |
| Luteal phase | 22 | 16.2 | 5.6 | 9.5–34.0 |
| Menstrual phase | 16 | 24.2 | 9.0 | 13.3–50.9 |
| Menopause | 16 | 12.1 | 3.4 | 6.9–18.0 |

### *Serum CA125 levels of patients with lung diseases*

Ninety-five patients with lung cancer and 43 patients with benign lung diseases were examined. The stages of lung cancer were decided according to TNM classification of the Union Internationale Contre le Cancer. The clinical features are summarised in Tables II and III. Increased serum levels of CA125 were observed in 14 out of 27 patients with squamous cell cancer (37.8%), in 14 out of 35 with adenocarcinoma (30.0%) and in nine out of 15 with small cell cancer (60.0%). These values were significant compared with that of normal subjects ($P<0.01$). Serum CA125 levels remained below the cut-off level in the patients with large cell lung cancer (Figure 2). Like other tumour markers, the serum CA125 level was high in the advanced stages of the disease. There was only one early-stage patient who showed an elevated serum CA125 level and he developed brain metastases 7 months later. It was found that most patients with pleural effusions or ascites had increased serum CA125 levels (11 out of 12), but 42.9% of patients in stages 3 and 4 without pleural effusions or ascites still had increased serum CA125 levels (squamous cell carcinoma 41.7%, adenocarcinoma 46.2% and small cell carcinoma 66.7%).

In contrast to lung cancer, only five out of 43 patients with benign lung diseases had increased serum CA125 levels (11.6%, normal *vs* benign lung diseases; $P<0.05$), and the degree of increase was slight (mean ± s.d.; 12.7 ± 13.6). Increased levels were found in one patient with sarcoidosis, three with primary interstitial pneumonitis, and one with silicosis, and pleural effusions was not present in these patients.

**Table II** Clinical features of patients with lung cancer

| | Type of lung cancer | | | |
|---|---|---|---|---|
| | SCC | Adeno | SCLC | LCLC |
| Total cases | 37 | 35 | 15 | 8 |
| Male | 35 | 22 | 14 | 7 |
| Female | 2 | 13 | 1 | 1 |
| Age median | 71 | 62 | 62 | 57 |
| Range | 50–79 | 25–79 | 49–80 | 33–75 |
| Stage 1 | 9 | 7 | 1 | 1 |
| 2 | 2 | 6 | 1 | 0 |
| 3 | 20 | 10 | 7 | 1 |
| 4 | 6 | 12 | 6 | 6 |
| With pleural effusion or ascites | 2 | 9 | 1 | 0 |

SCC, squamous cell carcinoma; adeno, adenocarcinoma; SCLC, small cell lung cancer; LCLC, large cell lung cancer.

**Table III** Benign lung diseases

| Diagnosis | Number of patients |
|---|---|
| Sarcoidosis | 10 |
| Pulmonary tuberculosis | 9 |
| Interstitial pneumonitis | 4 |
| Pulmonary fibrosis | 5 |
| Silicosis | 1 |
| Asthma | 1 |
| Wegener's granuloma | 1 |
| Diffuse panbronchiolitis | 2 |
| Bronchitis or pneumonia | 5 |
| Bronchiectasis | 3 |
| Abscess | 1 |
| Chronic obstructive lung disease | 1 |

### *Relationship of CA125 level to prognosis*

We compared the survival rate of patients with respect to the CA125 level, the presence of pleural effusion, staging, and histology. As shown in Figure 3, the survival curve of CA125 + (serum CA125 level > cut-off level) patients was markedly worse than that of CA125 − (serum CA125 level ⩽ cut-off level) patients. As almost all CA125 + patients were in the advanced stages, a further evaluation was performed comparing stage 3 and 4 patients. Among the patients with stage 3 and 4 lung cancer, the survival curve of CA125 + cases was significantly worse, and no CA125 + case survived beyond 2 years. The overall survival curve in stage 4 cancer was worse than in stage 3 cancer, but survival beyond 1.5 years did not differ significantly (Figure 4). The existence of a pleural effusion did not affect the survival rate (Figure 5; $P<0.05$). Although squamous cell cancer was associated with better survival than small cell cancer ($P<0.05$), the other types of lung cancer did not differ significantly from each other (Figure 6).

**Figure 2** Serum CA125 levels of patients with lung diseases. Closed circles indicate males and open circles indicate females. Large circles indicate patients with pleural effusion or ascites.

**Figure 3** Survival curves of patients with lung cancer depended on their serum CA125 level. **a**, all cases examined (CA125 + *vs* CA125 −, $P<0.01$). **b**, patients in stages 3 and 4 (CA125 + *vs* CA125 −, $P<0.05$).

**Figure 4** Survival curves of patients with lung cancer depended on their stages (stage 4 *vs* stage 3, $P < 0.05$).

**Figure 5** Survival curves of patients with stage 3 and 4 lung cancer with or without pleural effusion (effusion + *vs* effusion −, $P > 0.05$).

**Figure 6** Survival curves of patients with stage 3 and 4 lung cancer based on its histological diagnosis (squamous cell lung cancer *vs* small cell lung cancer, $P < 0.05$).

### Discussion

We evaluated the serum CA125 levels of patients with lung cancer using a one-step immunoradiometric assay. Although previous reports have revealed close association between ovarian tumours and serum CA125 levels, there have been few papers which show the significance of CA125 in lung cancer. In this study, by setting different cut-off values for males and females, we found a high positive rate in advanced lung cancer. Furthermore, increased serum CA125 levels were observed in patients with a poor prognosis.

It is notable that 38% of patients with lung cancer had elevated serum CA125 levels, and that if one looks at stage 3 and 4 patients, the marker was present in 49% of those with squamous cell carcinoma (42% of those without effusions), 63% of those with adenocarcinoma (46% of those without effusions) and 69% of those with small call carcinoma (67% of those without effusions). CA125 is an antigen of ovarian adenocarcinoma cells, and recently it has been shown to exist on the ectodermal cells of the peritoneum and pleura (Kabawat *et al.*, 1983*b*). This may explain why most patients with pleural effusions or ascites had increased serum CA125 levels, but the marker was still present in relatively high percent of patients without effusions (42% of those with squamous cell carcinoma, 46% of those with adenocarcinoma and 67% of those with small cell carcinoma). Immunohistological examination demonstrated CA125 antigen in the lung adenocarcinoma but failed to demonstrate in the other types of lung cancer (Mutsuoka *et al.*, 1987). Although the site of production of CA125 in advanced stages is not clarified, we consider that those patients who were CA125 + without pleural effusions may have had microscopic invasion of the pleura and the serum level of this marker correlate to the stage of the disease. The cell type variations may be the area for a further study.

We found that an increased serum CA125 level indicated a bad prognostic factor for lung cancer. As CA125 + patients were more often stage 4 and more often had small cell cancer, the survival curves were compared between stages 3 and 4, among histological types, and between patients with and without pleural effusion. Although there were some differences between stages 3 and 4 and between squamous cell and small cell cancer, the CA125 levels (positive or not) was best correlated with survival time.

We conclude that CA125 is a good indicator of disease extent and correlates with the prognosis.

The authors thank Ms Mariko Ata for her technical assistant.

### References

BAST, R.C., FEENEY, M., LAZARUS, H., NADLER, L.M., COLVIN, R.B. & KNAPP, R.C. (1981). Reactivity of a monoclonal antibody with human ovarian carcinoma. *J. Clin. Invest.*, **68**, 1331.

BAST, R.C., KLUG, T.L., ST JOHN, E. & 9 others (1983). A radioimmunoassay using a monoclonal antibody to monitor the course of epithelial ovarian cancer. *N. Engl. J. Med.*, **309**, 883.

BUAMAH, P.K., CORNELL, C., SKILLEN, A.W., CANTWELL, B.M.J. & HARRIS, A.L. (1987). Initial assessment of tumor-associated antigen CA-125 in patients with ovarian, cervical, and testicular tumors. *Clin. Chem.*, **33**, 1124.

FLEUREN, G.J., NAP, M., AALDERS, J.G., TRIMBOS, J.B. & BRUIJN, H.N.A. (1987). Explanation of the limited correlation between tumor CA125 content and serum CA125 antigen levels in patients with ovarian tumors. *Cancer*, **60**, 2437.

KABAWAT, S.E., BAST, R.C., WELCH, W.R., KNAPP, R.C. & COLVIN, R.B. (1983*a*). Immunopathologic characterization of a monoclonal antibody that recognizes common surface antigens of human ovarian tumors of serous, endometrioid, and clear cell types. *Am. J. Clin. Pathol.*, **79**, 98.

KABAWAT, S.E., BAST, R.C., BHAN, A.K., WELCH, W.R., KNAPP, R.C. & COLVIN, R.B. (1983*b*). Tissue distribution of a coelomic-epithelium-related antigen recognized by the monoclonal antibody OC125. *Int. J. Pathol.*, **2**, 275.

KUNIMATSU, M., ENDO, K., NAKASHIMA, T. & 14 others (1988). Development of new immunoradiometric assay for CA125 antigen using two monoclonal antibodies produced by immunizing lung cancer cells. *Ann. Nucl. Med.*, **2**, 73.

MATSUOKA, Y., NAKASHIMA, T., ENDO, K. & 7 others (1987). Recognition of ovarian cancer antigen CA125 by murine monoclonal antibody produced by immunization of lung cancer cells. *Cancer Res.*, **47**, 6335.

TAKAHASHI, K., NAGATA, H., YAMANE, Y. & 4 others (1986). Clinical usefulness of serum CA125 in patients with endometriosis. *Shimane. J. Med.*, **9**, 82.


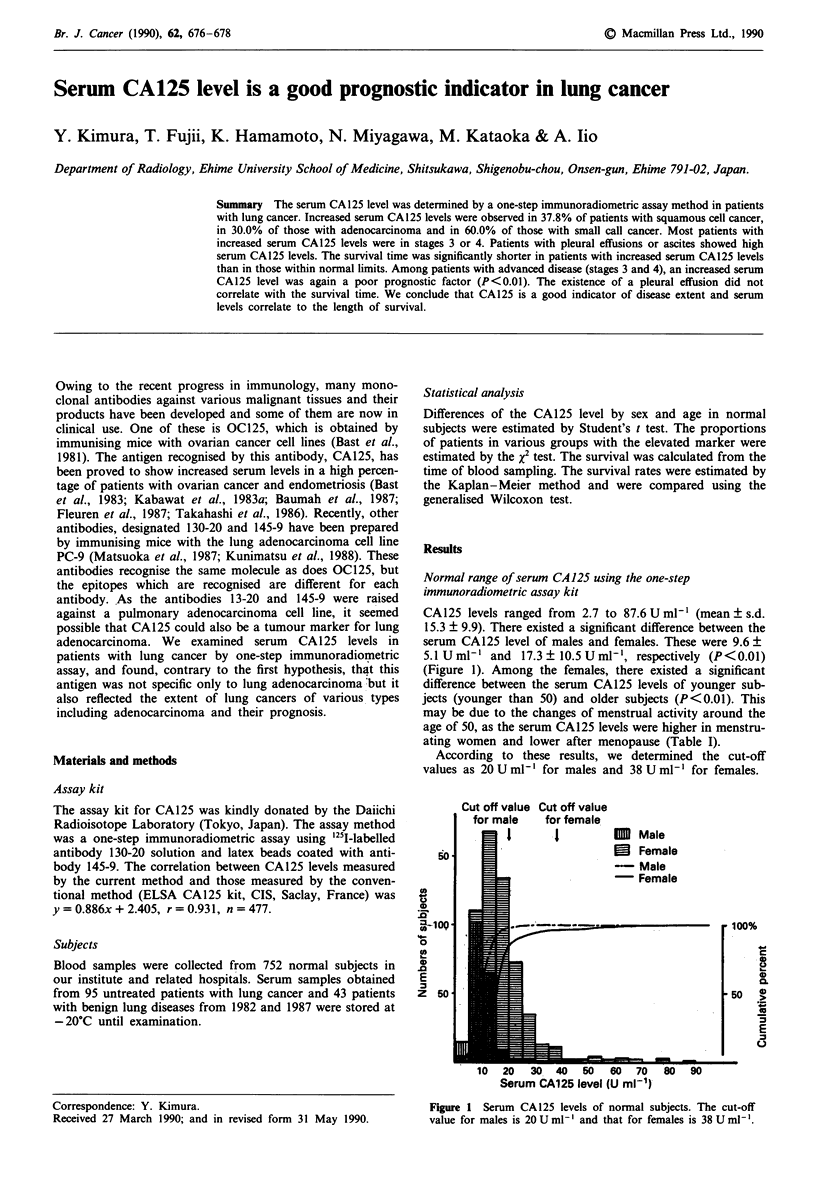

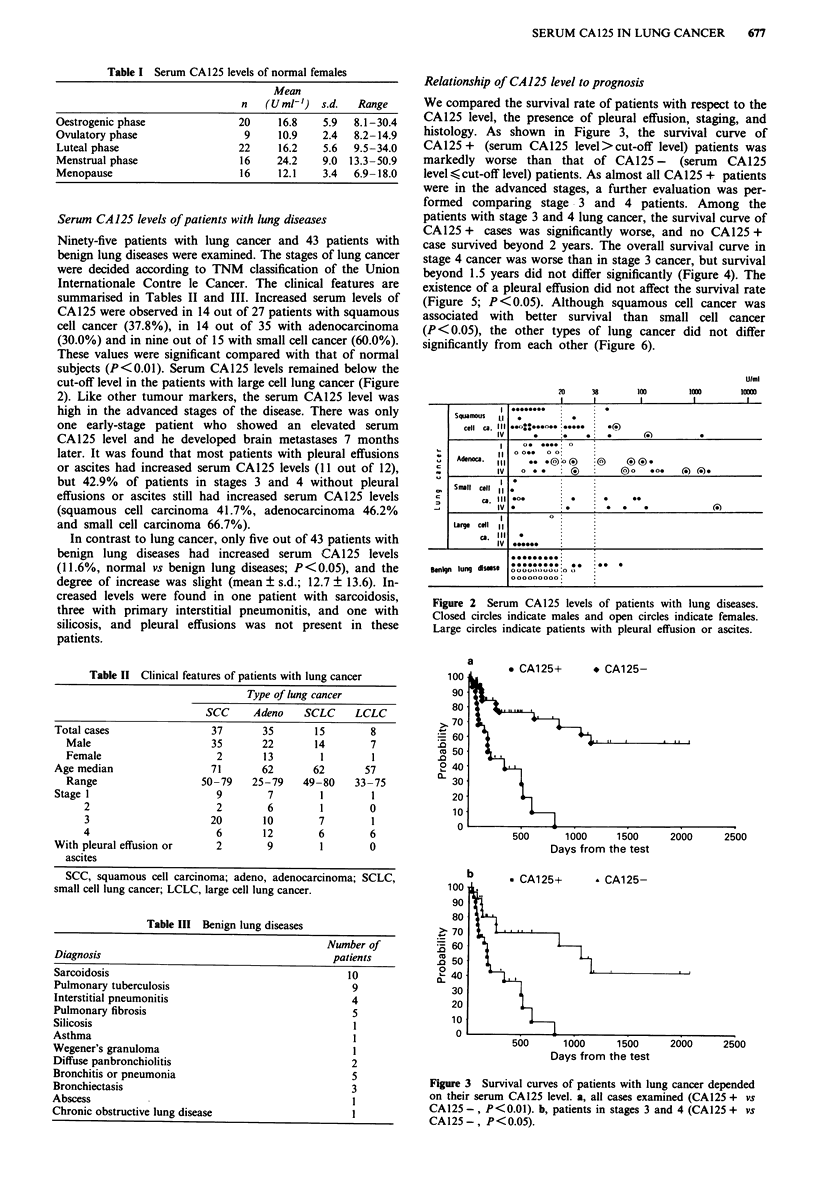

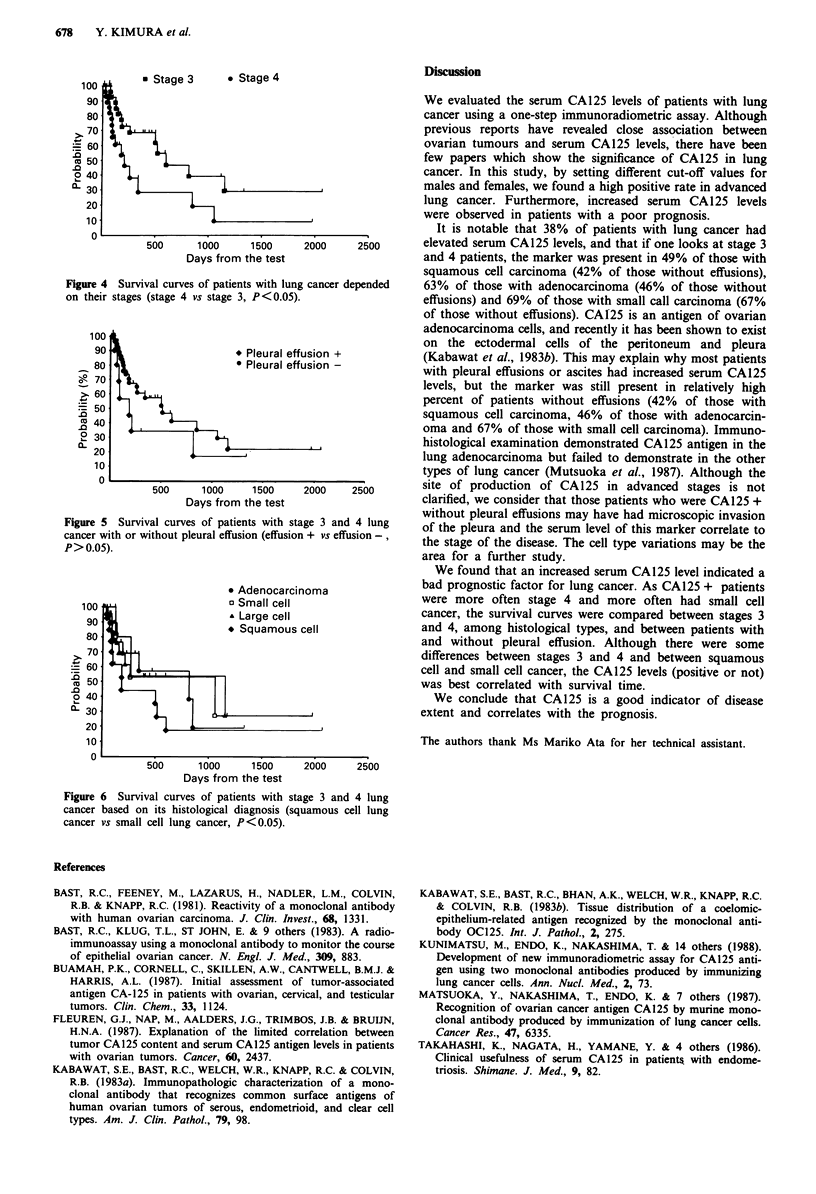


## References

[OCR_00478] Bast R. C., Feeney M., Lazarus H., Nadler L. M., Colvin R. B., Knapp R. C. (1981). Reactivity of a monoclonal antibody with human ovarian carcinoma.. J Clin Invest.

[OCR_00483] Bast R. C., Klug T. L., St John E., Jenison E., Niloff J. M., Lazarus H., Berkowitz R. S., Leavitt T., Griffiths C. T., Parker L. (1983). A radioimmunoassay using a monoclonal antibody to monitor the course of epithelial ovarian cancer.. N Engl J Med.

[OCR_00488] Buamah P. K., Cornell C., Skillen A. W., Cantwell B. M., Harris A. L. (1987). Initial assessment of tumor-associated antigen CA-125 in patients with ovarian, cervical, and testicular tumors.. Clin Chem.

[OCR_00494] Fleuren G. J., Nap M., Aalders J. G., Trimbos J. B., de Bruijn H. W. (1987). Explanation of the limited correlation between tumor CA 125 content and serum CA 125 antigen levels in patients with ovarian tumors.. Cancer.

[OCR_00507] Kabawat S. E., Bast R. C., Bhan A. K., Welch W. R., Knapp R. C., Colvin R. B. (1983). Tissue distribution of a coelomic-epithelium-related antigen recognized by the monoclonal antibody OC125.. Int J Gynecol Pathol.

[OCR_00500] Kabawat S. E., Bast R. C., Welch W. R., Knapp R. C., Colvin R. B. (1983). Immunopathologic characterization of a monoclonal antibody that recognizes common surface antigens of human ovarian tumors of serous, endometrioid, and clear cell types.. Am J Clin Pathol.

[OCR_00515] Matsuoka Y., Nakashima T., Endo K., Yoshida T., Kunimatsu M., Sakahara H., Koizumi M., Nakagawa T., Yamaguchi N., Torizuka K. (1987). Recognition of ovarian cancer antigen CA125 by murine monoclonal antibody produced by immunization of lung cancer cells.. Cancer Res.

